# Supplementary material for: COZOID: contact zone identifier for visual analysis of protein-protein interactions
Source: BMC Bioinformatics. 2018 Apr 6;19:125. doi: 10.1186/s12859-018-2113-6 (PMC5889581; doi:10.1186/s12859-018-2113-6)
Supplement: Supplementary file 2 — Software build. Executable binary file of the software tool. (ZIP 118,784 kb) [file 12859_2018_2113_MOESM2_ESM.zip › cozoid/platform/docs/testng-6.5.1-javadoc.zip!/org/testng/TestNGAntTask.html]

TestNGAntTask (TestNG)


---


|  |  |  |
| --- | --- | --- |
| **Overview** \| **Package** \| **Class** \| **Tree** \| **Deprecated** \| **Index** \| **Help** | |  |
| **PREV CLASS**   **NEXT CLASS** | **FRAMES**    **NO FRAMES**     **All Classes** |  |
| SUMMARY: NESTED \| FIELD \| CONSTR \| METHOD | DETAIL: FIELD \| CONSTR \| METHOD |  |


---


## org.testng Class TestNGAntTask

```
java.lang.Object
  org.apache.tools.ant.ProjectComponent
      org.apache.tools.ant.Task
          org.testng.TestNGAntTask
```

**All Implemented Interfaces:**: java.lang.Cloneable

---

``` public class TestNGAntTask extends org.apache.tools.ant.Task ```

TestNG settings:

- classfileset (inner)
- classfilesetref (attribute)
- xmlfileset (inner)
- xmlfilesetref (attribute)
- enableAssert (attribute)
- excludedGroups (attribute)
- groups (attribute)
- junit (attribute)
- listener (attribute)
- outputdir (attribute)
- parallel (attribute)
- reporter (attribute)
- sourcedir (attribute)
- sourcedirref (attribute)
- suitename (attribute)
- suiterunnerclass (attribute)
- target (attribute)
- testjar (attribute)
- testname (attribute)
- threadcount (attribute)
- dataproviderthreadcount (attribute)
- verbose (attribute)
- testrunfactory (attribute)
- configFailurepolicy (attribute)
- randomizeSuites (attribute)
- methodselectors (attribute)

Ant settings:

- classpath (inner)
- classpathref (attribute)
- jvm (attribute)
- workingDir (attribute)
- env (inner)
- sysproperty (inner)
- propertyset (inner)
- jvmarg (inner)
- timeout (attribute)
- haltonfailure (attribute)
- onHaltTarget (attribute)
- failureProperty (attribute)
- haltonFSP (attribute)
- FSPproperty (attribute)
- haltonskipped (attribute)
- skippedProperty (attribute)
- testRunnerFactory (attribute)

Debug information:

- dumpCommand (boolean)
- dumpEnv (boolean)
- dumpSys (boolean)

**Author:**
:   Alexandru Popescu, Cedric Beust, Lukas Jungmann

---

| **Nested Class Summary** | |
| --- | --- |
| `static class` | `TestNGAntTask.Mode` |
| `protected static class` | `TestNGAntTask.TestNGLogSH` |


| **Field Summary** | |
| --- | --- |
| `protected  boolean` | `m_assertEnabled` |
| `protected  java.util.List<org.apache.tools.ant.types.ResourceCollection>` | `m_classFilesets` |
| `protected  java.lang.String` | `m_configFailurePolicy` |
| `protected  java.lang.String` | `m_dataproviderthreadCount` |
| `protected  boolean` | `m_dump`             True if the temporary file created by the Ant Task for command line parameters to TestNG should be preserved after execution. |
| `protected  org.apache.tools.ant.types.Environment` | `m_environment` |
| `protected  java.lang.String` | `m_excludedGroups` |
| `protected  java.lang.String` | `m_failurePropertyName` |
| `protected  java.lang.String` | `m_fspPropertyName` |
| `protected  boolean` | `m_haltOnFailure` |
| `protected  boolean` | `m_haltOnFSP` |
| `protected  boolean` | `m_haltOnSkipped` |
| `protected  java.lang.String` | `m_includedGroups` |
| `protected  org.apache.tools.ant.types.CommandlineJava` | `m_javaCommand` |
| `protected  java.lang.String` | `m_mainClass`             The suite runner name (defaults to TestNG.class.getName(). |
| `protected  java.lang.String` | `m_onHaltTarget` |
| `protected  java.io.File` | `m_outputDir` |
| `protected  java.lang.String` | `m_parallelMode` |
| `protected  java.lang.Boolean` | `m_randomizeSuites` |
| `protected  java.lang.String` | `m_skippedPropertyName` |
| `protected  java.io.File` | `m_testjar` |
| `protected  java.lang.String` | `m_testRunnerFactory` |
| `protected  java.lang.String` | `m_threadCount` |
| `java.lang.String` | `m_useDefaultListeners` |
| `protected  java.io.File` | `m_workingDir` |
| `protected  java.util.List<org.apache.tools.ant.types.ResourceCollection>` | `m_xmlFilesets` |

**Fields inherited from class org.apache.tools.ant.Task**

`target, taskName, taskType, wrapper`

**Fields inherited from class org.apache.tools.ant.ProjectComponent**

`description, location, project`


**Constructor Summary**

`TestNGAntTask()`


| **Method Summary** | |
| --- | --- |
| `protected  void` | `actOnResult(int exitValue, boolean wasKilled)` |
| `void` | `addClassfileset(org.apache.tools.ant.types.FileSet fs)` |
| `void` | `addConfiguredPropertySet(org.apache.tools.ant.types.PropertySet sysPropertySet)`             Add the referenced property set as system properties for the TestNG JVM. |
| `void` | `addConfiguredReporter(ReporterConfig reporterConfig)` |
| `void` | `addEnv(org.apache.tools.ant.types.Environment.Variable var)`             Adds an environment variable; used when forking. |
| `void` | `addSysproperty(org.apache.tools.ant.types.Environment.Variable sysp)` |
| `void` | `addXmlfileset(org.apache.tools.ant.types.FileSet fs)` |
| `org.apache.tools.ant.types.Path` | `createBootclasspath()`             Adds a path to the bootclasspath. |
| `org.apache.tools.ant.types.Path` | `createClasspath()`             Adds path to classpath used for tests. |
| `org.apache.tools.ant.types.Commandline.Argument` | `createJvmarg()` |
| `protected  org.apache.tools.ant.taskdefs.ExecuteWatchdog` | `createWatchdog()` |
| `void` | `execute()`             Launches TestNG in a new JVM. |
| `protected  int` | `executeAsForked(org.apache.tools.ant.types.CommandlineJava cmd, org.apache.tools.ant.taskdefs.ExecuteWatchdog watchdog)`             Executes the command line as a new process. |
| `protected  org.apache.tools.ant.types.CommandlineJava` | `getJavaCommand()`             Creates or returns the already created `CommandlineJava`. |
| `protected  java.util.List<java.lang.String>` | `getSuiteFileNames()` |
| `protected  void` | `handleOutput(java.lang.String output)` |
| `void` | `setClassfilesetRef(org.apache.tools.ant.types.Reference ref)` |
| `void` | `setClasspath(org.apache.tools.ant.types.Path s)`             Set the classpath to be used when running the Java class |
| `void` | `setClasspathRef(org.apache.tools.ant.types.Reference r)`             Classpath to use, by reference. |
| `void` | `setConfigFailurePolicy(java.lang.String failurePolicy)` |
| `void` | `setDataProviderThreadCount(java.lang.String dataproviderthreadCount)` |
| `void` | `setDelegateCommandSystemProperties(boolean value)` |
| `void` | `setDumpCommand(boolean verbose)`             Sets the flag to log the command line. |
| `void` | `setDumpEnv(boolean verbose)`             Sets the flag to write on `System.out` the Ant Environment properties. |
| `void` | `setDumpSys(boolean verbose)`             Sets te flag to write on `System.out` the system properties. |
| `void` | `setEnableAssert(boolean flag)` |
| `void` | `setExcludedGroups(java.lang.String groups)` |
| `void` | `setFailureProperty(java.lang.String propertyName)` |
| `void` | `setFSPProperty(java.lang.String propertyName)` |
| `void` | `setGroups(java.lang.String groups)` |
| `void` | `setHaltonfailure(boolean value)` |
| `void` | `setHaltonFSP(boolean value)` |
| `void` | `setHaltonskipped(boolean value)` |
| `void` | `setJUnit(boolean value)` |
| `void` | `setJvm(java.lang.String jvm)`             Sets a particular JVM to be used. |
| `void` | `setListener(java.lang.String listener)`             **Deprecated.** *Use "listeners"* |
| `void` | `setListeners(java.lang.String listeners)` |
| `void` | `setMethods(java.lang.String methods)` |
| `void` | `setMethodSelectors(java.lang.String methodSelectors)` |
| `void` | `setMode(TestNGAntTask.Mode mode)` |
| `void` | `setObjectFactory(java.lang.String className)` |
| `void` | `setOnHaltTarget(java.lang.String targetName)` |
| `void` | `setOutputDir(java.io.File dir)`             Sets the test output directory |
| `void` | `setParallel(java.lang.String parallel)` |
| `void` | `setRandomizeSuites(java.lang.Boolean randomizeSuites)` |
| `void` | `setReporter(java.lang.String listener)` |
| `void` | `setSkipFailedInvocationCounts(boolean skip)` |
| `void` | `setSkippedProperty(java.lang.String propertyName)` |
| `void` | `setSuiteName(java.lang.String s)`             Sets the suite name |
| `void` | `setSuiteRunnerClass(java.lang.String s)`             Sets the suite runner class to invoke |
| `void` | `setSuiteThreadPoolSize(java.lang.Integer n)` |
| `void` | `setTestJar(java.io.File s)`             Sets the test jar |
| `void` | `setTestName(java.lang.String s)`             Sets the test name |
| `void` | `setTestNames(java.lang.String testNames)` |
| `void` | `setTestRunnerFactory(java.lang.String testRunnerFactory)` |
| `void` | `setThreadCount(java.lang.String threadCount)` |
| `void` | `setTimeout(java.lang.Integer value)`             Set the timeout value (in milliseconds). |
| `void` | `setUseDefaultListeners(java.lang.String f)` |
| `void` | `setVerbose(java.lang.Integer verbose)` |
| `void` | `setWorkingDir(java.io.File workingDir)`             The directory to invoke the VM in. |
| `void` | `setXmlfilesetRef(org.apache.tools.ant.types.Reference ref)` |
| `void` | `setXmlPathInJar(java.lang.String path)` |
| `protected  void` | `validateOptions()` |

**Methods inherited from class org.apache.tools.ant.Task**

`bindToOwner, getOwningTarget, getRuntimeConfigurableWrapper, getTaskName, getTaskType, getWrapper, handleErrorFlush, handleErrorOutput, handleFlush, handleInput, init, isInvalid, log, log, log, log, maybeConfigure, perform, reconfigure, setOwningTarget, setRuntimeConfigurableWrapper, setTaskName, setTaskType`

**Methods inherited from class org.apache.tools.ant.ProjectComponent**

`clone, getDescription, getLocation, getProject, setDescription, setLocation, setProject`

**Methods inherited from class java.lang.Object**

`equals, finalize, getClass, hashCode, notify, notifyAll, toString, wait, wait, wait`

**Field Detail**

### m\_javaCommand

```
protected org.apache.tools.ant.types.CommandlineJava m_javaCommand
```

---


### m\_xmlFilesets

```
protected java.util.List<org.apache.tools.ant.types.ResourceCollection> m_xmlFilesets
```

---


### m\_classFilesets

```
protected java.util.List<org.apache.tools.ant.types.ResourceCollection> m_classFilesets
```

---


### m\_outputDir

```
protected java.io.File m_outputDir
```

---


### m\_testjar

```
protected java.io.File m_testjar
```

---


### m\_workingDir

```
protected java.io.File m_workingDir
```

---


### m\_testRunnerFactory

```
protected java.lang.String m_testRunnerFactory
```

---


### m\_environment

```
protected org.apache.tools.ant.types.Environment m_environment
```

---


### m\_mainClass

```
protected java.lang.String m_mainClass
```

:   The suite runner name (defaults to TestNG.class.getName().

---


### m\_dump

```
protected boolean m_dump
```

:   True if the temporary file created by the Ant Task for command line parameters
    to TestNG should be preserved after execution.

---


### m\_assertEnabled

```
protected boolean m_assertEnabled
```

---


### m\_haltOnFailure

```
protected boolean m_haltOnFailure
```

---


### m\_onHaltTarget

```
protected java.lang.String m_onHaltTarget
```

---


### m\_failurePropertyName

```
protected java.lang.String m_failurePropertyName
```

---


### m\_haltOnSkipped

```
protected boolean m_haltOnSkipped
```

---


### m\_skippedPropertyName

```
protected java.lang.String m_skippedPropertyName
```

---


### m\_haltOnFSP

```
protected boolean m_haltOnFSP
```

---


### m\_fspPropertyName

```
protected java.lang.String m_fspPropertyName
```

---


### m\_includedGroups

```
protected java.lang.String m_includedGroups
```

---


### m\_excludedGroups

```
protected java.lang.String m_excludedGroups
```

---


### m\_parallelMode

```
protected java.lang.String m_parallelMode
```

---


### m\_threadCount

```
protected java.lang.String m_threadCount
```

---


### m\_dataproviderthreadCount

```
protected java.lang.String m_dataproviderthreadCount
```

---


### m\_configFailurePolicy

```
protected java.lang.String m_configFailurePolicy
```

---


### m\_randomizeSuites

```
protected java.lang.Boolean m_randomizeSuites
```

---


### m\_useDefaultListeners

```
public java.lang.String m_useDefaultListeners
```


**Constructor Detail**

### TestNGAntTask

```
public TestNGAntTask()
```


**Method Detail**

### setParallel

```
public void setParallel(java.lang.String parallel)
```

---


### setThreadCount

```
public void setThreadCount(java.lang.String threadCount)
```

---


### setDataProviderThreadCount

```
public void setDataProviderThreadCount(java.lang.String dataproviderthreadCount)
```

---


### setUseDefaultListeners

```
public void setUseDefaultListeners(java.lang.String f)
```

---


### setHaltonfailure

```
public void setHaltonfailure(boolean value)
```

---


### setOnHaltTarget

```
public void setOnHaltTarget(java.lang.String targetName)
```

---


### setFailureProperty

```
public void setFailureProperty(java.lang.String propertyName)
```

---


### setHaltonskipped

```
public void setHaltonskipped(boolean value)
```

---


### setSkippedProperty

```
public void setSkippedProperty(java.lang.String propertyName)
```

---


### setHaltonFSP

```
public void setHaltonFSP(boolean value)
```

---


### setFSPProperty

```
public void setFSPProperty(java.lang.String propertyName)
```

---


### setDelegateCommandSystemProperties

```
public void setDelegateCommandSystemProperties(boolean value)
```

---


### setDumpCommand

```
public void setDumpCommand(boolean verbose)
```

:   Sets the flag to log the command line. When verbose is set to true
    the command line parameters are stored in a temporary file stored
    in the user's default temporary file directory. The file created is
    prefixed with "testng".

---


### setDumpEnv

```
public void setDumpEnv(boolean verbose)
```

:   Sets the flag to write on `System.out` the Ant
    Environment properties.

    :   **Parameters:**: `verbose` - true for printing

---


### setDumpSys

```
public void setDumpSys(boolean verbose)
```

:   Sets te flag to write on `System.out` the system properties.

    :   **Parameters:**: `verbose` - true for dumping the info

---


### setEnableAssert

```
public void setEnableAssert(boolean flag)
```

---


### setWorkingDir

```
public void setWorkingDir(java.io.File workingDir)
```

:   The directory to invoke the VM in.

    :   **Parameters:**: `workingDir` - the directory to invoke the JVM from.

---


### setJvm

```
public void setJvm(java.lang.String jvm)
```

:   Sets a particular JVM to be used. Default is 'java' and is solved
    by `Runtime.exec()`.

    :   **Parameters:**: `jvm` - the new jvm

---


### setTimeout

```
public void setTimeout(java.lang.Integer value)
```

:   Set the timeout value (in milliseconds).

    If the tests are running for more than this value, the tests
    will be canceled.

    :   **Parameters:**: `value` - the maximum time (in milliseconds) allowed before declaring the test as 'timed-out'

---


### createJvmarg

```
public org.apache.tools.ant.types.Commandline.Argument createJvmarg()
```

---


### addSysproperty

```
public void addSysproperty(org.apache.tools.ant.types.Environment.Variable sysp)
```

---


### addEnv

```
public void addEnv(org.apache.tools.ant.types.Environment.Variable var)
```

:   Adds an environment variable; used when forking.

---


### createClasspath

```
public org.apache.tools.ant.types.Path createClasspath()
```

:   Adds path to classpath used for tests.

    :   **Returns:**: reference to the classpath in the embedded java command line

---


### createBootclasspath

```
public org.apache.tools.ant.types.Path createBootclasspath()
```

:   Adds a path to the bootclasspath.

    :   **Returns:**: reference to the bootclasspath in the embedded java command line

---


### setClasspath

```
public void setClasspath(org.apache.tools.ant.types.Path s)
```

:   Set the classpath to be used when running the Java class

    :   **Parameters:**: `s` - an Ant Path object containing the classpath.

---


### setClasspathRef

```
public void setClasspathRef(org.apache.tools.ant.types.Reference r)
```

:   Classpath to use, by reference.

    :   **Parameters:**: `r` - a reference to an existing classpath

---


### addXmlfileset

```
public void addXmlfileset(org.apache.tools.ant.types.FileSet fs)
```

---


### setXmlfilesetRef

```
public void setXmlfilesetRef(org.apache.tools.ant.types.Reference ref)
```

---


### addClassfileset

```
public void addClassfileset(org.apache.tools.ant.types.FileSet fs)
```

---


### setClassfilesetRef

```
public void setClassfilesetRef(org.apache.tools.ant.types.Reference ref)
```

---


### setTestNames

```
public void setTestNames(java.lang.String testNames)
```

---


### setSuiteRunnerClass

```
public void setSuiteRunnerClass(java.lang.String s)
```

:   Sets the suite runner class to invoke

    :   **Parameters:**: `s` - the name of the suite runner class

---


### setSuiteName

```
public void setSuiteName(java.lang.String s)
```

:   Sets the suite name

    :   **Parameters:**: `s` - the name of the suite

---


### setTestName

```
public void setTestName(java.lang.String s)
```

:   Sets the test name

    :   **Parameters:**: `s` - the name of the test

---


### setJUnit

```
public void setJUnit(boolean value)
```

---


### setMode

```
public void setMode(TestNGAntTask.Mode mode)
```

---


### setOutputDir

```
public void setOutputDir(java.io.File dir)
```

:   Sets the test output directory

    :   **Parameters:**: `dir` - the name of directory

---


### setTestJar

```
public void setTestJar(java.io.File s)
```

:   Sets the test jar

    :   **Parameters:**: `s` - the name of test jar

---


### setGroups

```
public void setGroups(java.lang.String groups)
```

---


### setExcludedGroups

```
public void setExcludedGroups(java.lang.String groups)
```

---


### setVerbose

```
public void setVerbose(java.lang.Integer verbose)
```

---


### setReporter

```
public void setReporter(java.lang.String listener)
```

---


### setObjectFactory

```
public void setObjectFactory(java.lang.String className)
```

---


### setTestRunnerFactory

```
public void setTestRunnerFactory(java.lang.String testRunnerFactory)
```

---


### setSuiteThreadPoolSize

```
public void setSuiteThreadPoolSize(java.lang.Integer n)
```

---


### setListener

```
@Deprecated
public void setListener(java.lang.String listener)
```

:   **Deprecated.** *Use "listeners"*

---


### setListeners

```
public void setListeners(java.lang.String listeners)
```

---


### setMethodSelectors

```
public void setMethodSelectors(java.lang.String methodSelectors)
```

---


### setConfigFailurePolicy

```
public void setConfigFailurePolicy(java.lang.String failurePolicy)
```

---


### setRandomizeSuites

```
public void setRandomizeSuites(java.lang.Boolean randomizeSuites)
```

---


### setMethods

```
public void setMethods(java.lang.String methods)
```

---


### execute

```
public void execute()
             throws org.apache.tools.ant.BuildException
```

:   Launches TestNG in a new JVM.

    :   **Overrides:**: `execute` in class `org.apache.tools.ant.Task`
    :   **Throws:**: `org.apache.tools.ant.BuildException`

---


### getSuiteFileNames

```
protected java.util.List<java.lang.String> getSuiteFileNames()
```

:   **Returns:**: the list of the XML file names. This method can be overridden by subclasses.

---


### actOnResult

```
protected void actOnResult(int exitValue,
                           boolean wasKilled)
```

---


### executeAsForked

```
protected int executeAsForked(org.apache.tools.ant.types.CommandlineJava cmd,
                              org.apache.tools.ant.taskdefs.ExecuteWatchdog watchdog)
```

:   Executes the command line as a new process.

    :   **Parameters:**: `cmd` - the command to execute: `watchdog` - **Returns:**: the exit status of the subprocess or INVALID.

---


### getJavaCommand

```
protected org.apache.tools.ant.types.CommandlineJava getJavaCommand()
```

:   Creates or returns the already created `CommandlineJava`.

---


### createWatchdog

```
protected org.apache.tools.ant.taskdefs.ExecuteWatchdog createWatchdog()
```

:   **Returns:**: null if there is no timeout value, otherwise the watchdog instance. **Throws:**: `org.apache.tools.ant.BuildException` - under unspecified circumstances **Since:** : Ant 1.2

---


### validateOptions

```
protected void validateOptions()
                        throws org.apache.tools.ant.BuildException
```

:   **Throws:**: `org.apache.tools.ant.BuildException`

---


### addConfiguredReporter

```
public void addConfiguredReporter(ReporterConfig reporterConfig)
```

---


### setSkipFailedInvocationCounts

```
public void setSkipFailedInvocationCounts(boolean skip)
```

---


### setXmlPathInJar

```
public void setXmlPathInJar(java.lang.String path)
```

---


### addConfiguredPropertySet

```
public void addConfiguredPropertySet(org.apache.tools.ant.types.PropertySet sysPropertySet)
```

:   Add the referenced property set as system properties for the TestNG JVM.

    :   **Parameters:**: `sysPropertySet` - A PropertySet of system properties.

---


### handleOutput

```
protected void handleOutput(java.lang.String output)
```

:   **Overrides:**: `handleOutput` in class `org.apache.tools.ant.Task`


---


|  |  |  |
| --- | --- | --- |
| **Overview** \| **Package** \| **Class** \| **Tree** \| **Deprecated** \| **Index** \| **Help** | |  |
| **PREV CLASS**   **NEXT CLASS** | **FRAMES**    **NO FRAMES**     **All Classes** |  |
| SUMMARY: NESTED \| FIELD \| CONSTR \| METHOD | DETAIL: FIELD \| CONSTR \| METHOD |  |


---
